# Supplementary material for: Stochastic modeling of intra- and inter-hospital transmission in Middle East respiratory syndrome outbreak
Source: PLoS One. 2025 Sep 26;20(9):e0332116. doi: 10.1371/journal.pone.0332116 (PMC12469155; doi:10.1371/journal.pone.0332116)
Supplement: S1 Text — This contains detailed explanation of methods for the research. (PDF) [file pone.0332116.s001.pdf]

S1 Text. for:
Stochastic Modeling of Intra- and Inter-Hospital Transmission in Middle East Respiratory Syndrome Outbreak

Youngsuk Ko[1], Jacob Lee[2], and Eunok Jung[3*]

1 Institute of Mathematical Sciences, Konkuk University, Seoul, Republic of Korea
2 Division of Infectious Diseases, Department of Internal Medicine, Kangnam Sacred Heart Hospital, Hallym University College of Medicine, Seoul, Republic of Korea
3 Department of Mathematics, Konkuk University, Seoul, Republic of Korea

* junge@konkuk.ac.kr

Before proceeding, we briefly introduce our study via Modeling Infectious Diseases in Healthcare Network (MInD-Healthcare) framework [1]:

Purpose and scope: Analysis of the risk of exceeding community quarantine levels upon the introduction of new infectious diseases and evaluation of the effectiveness of hospital-based interventions. Reflection of intra- and inter- hospital transmission following the initial introduction before community spread. Estimation of the transmission rate within hospitals based on epidemiological investigation data of hospital cluster infections during the 2015 MERS outbreak in Korea. Super-spreaders were not classified to observe general situations.

Entities, state variables, and scales: Entities: Local resident, medical staff, inpatient, hospital visitor. State variables: Susceptible, exposed, infectious, hospitalized, isolated, recovered. Scales: 650,000 residents in local, 36 hospitals with 9,305 medical staffs and 5,222 inpatients.

Initialization: A single local resident in the incubation stage arrives in the local community.

Process overview and scheduling: Outbreak is detected after a delay generated from data-fitted distribution once the index case is hospitalized. After outbreak detection, hospital visit becomes prohibited, and intra-hospital transmission rates are reduced by 59%.

Input data: Outbreak detection delay, intra-hospital intervention intensity for model simulation.

Agent interactions and organism: We assumed homogeneous mixing within hospital (or within local community), and the frequency-dependent disease transmission rate.

Stochasticity: Stochastic simulation using modified Gillespie algorithm was proceeded. Both of Markovian (nondelayed reaction, i.e., disease transmission) and non-Markovian (delayed reaction, i.e., disease progression) were considered.

Submodels: Detailed description of model formulation and parameters are described in **2 Model formulation**.

Model verification, calibration, and validation: To estimate the model parameters, we considered the first hospital-cluster infection in 2015, Korea. We formulated likelihood function using information from epidemiological investigation containing individual history. We used Metropolis-Hastings algorithm to estimate the posterior distribution of transmission rates within a hospital. We fitted individual based delay data (incubation period, duration from onset to isolation) assuming gamma

distribution. For the simulation results, we calculated averages and 95% credible intervals of simulation outputs using stochastic simulation algorithm. Lastly, we performed sensitivity analysis to investigate which factor is significant during an outbreak.

1 Data aggregation

In this study, data was aggregated from two sources regarding the spread of MERS in the Republic of Korea in 2015. First, data on the incubation period and the duration from symptom onset to confirmation of MERS cases was provided from Virlogeux's study [2]. This data was used in the section **Model formulation** to reflect the distribution of delays in the model. Additionally, data on the expected timing of the disease exposure and transmission for confirmed cases at Pyeongtaek St. Mary's Hospital was aggregated from Kim's study [3]. This data was utilized in the section **Parameter sampling** to estimate transmission rates.

2 Model formulation

Drawing upon an SEIR -type mathematical model, we formulated a comprehensive framework considering intra- and inter-hospital disease transmission. Within this model, we delineated six distinct epidemiological stages relevant to disease propagation: susceptible (S), exposed (E), infectious (I), hospitalized (H), isolated (Q), and recovered (R). Specifically, within the hospital context, hosts were further categorized into sub-groups: medical staff (denoted by subscript M), inpatients (P), and visitors (V). Notably, we did not differentiate superspreaders in this study. The flowchart depicted in Fig 1 visually represents the epidemiological process captured by our model. The superscript i denotes the hospital identifier. Solid lines represent infection transmission events following a Markovian process that disregards past events. The remaining dotted lines correspond to non-Markovian processes, considering past events, such as the incubation period after infection exposure (1, $\tau_{E\to I}$), the infectious period of visitors and local community hosts (2, $\tau_{I\to H}$), intra-hospital transmission period (3, $\tau_{I\to Q}$ and $\tau_{H\to Q}$), and isolation treatment duration (4, $\tau_{Q\to R}$). Both visitors and local residents undergo two delays: infection-to-hospital admission ($\tau_{I\to H}$) and admission-to-quarantine ($\tau_{I\to Q}$). Each delay is sampled from the same gamma distribution fitted to the observed time from symptom onset to isolation. In this study, we estimated these delays using gamma distributions based on data from confirmed patients and the MATLAB built-in function fitdist. Note that estimated parameters for the distributions are scale and shape parameters. These estimates were incorporated into the model simulation [2]. Fig 2 illustrates the fitted time delay distribution. We assumed a fixed isolation period of 28 days, while other time delays were generated from the following fitted distributions:

$$\tau_{E\to I} \sim \Gamma(4.4493, 1.5702),$$
$$\tau_{I\to H} = \tau_{H\to Q} \sim \Gamma(2.8118, 1.7996).$$

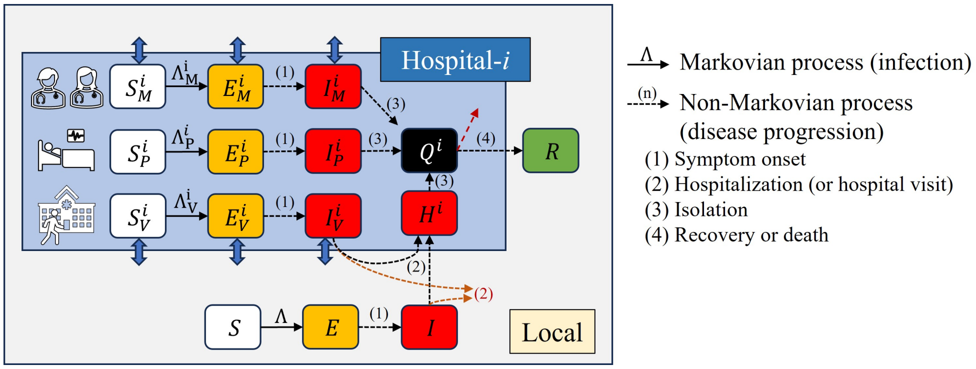

Fig 1. Flow diagram of Middle East Respiratory Syndrome outbreak model considering intra- and inter-hospital transmission.

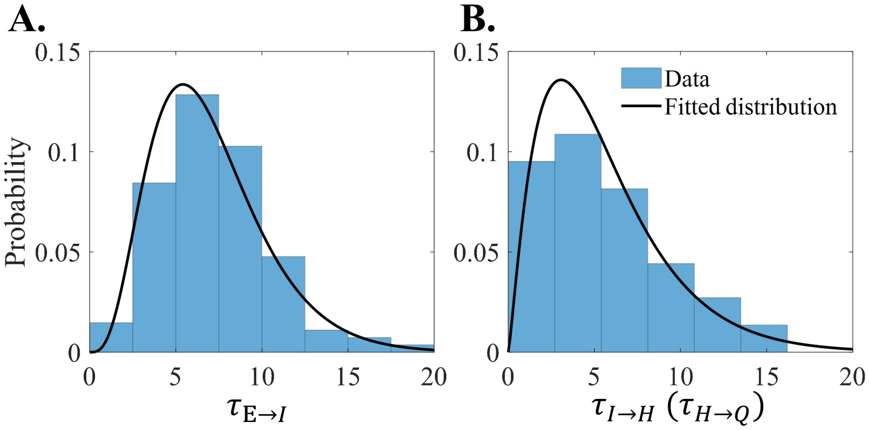

Fig 2. Distribution of time delay and fitted curve. Incubation period(A), Infectious period (B).

Both visitors and medical staff can transmit infections beyond the hospital premises. If visitors become infected, they are randomly assigned to one of the hospitals—potentially different from their original point of contact. To model this, we include the parameter a, defined as the inverse of the number of hospitals in our study ($a = 1/36$), which uniformly distributes incoming infected visitors across all facilities. For this study, we assume that there are no unreported cases. Asymptomatic cases fall within either stage I or H. The transmission rate in the community is denoted by β_L, and within hospitals the subscripts indicate the infector and infectee, respectively. For example, β_{PV} represents the transmission rate from inpatients to visitors. The model can be succinctly expressed using delay differential equations as follows:

$$
\begin{aligned}
\frac{dS}{dt} &= -S\frac{\beta_L\left(I+\sum_j\left(I_M^j+I_V^j\right)\right)}{N},\\
\frac{dE}{dt} &= S\frac{\beta_L\left(I+\sum_j\left(I_M^j+I_V^j\right)\right)}{N}-E\left(t-\tau_{E\to I}\right),\\
\frac{dI}{dt} &= E\left(t-\tau_{E\to I}\right)-I\left(t-\tau_{I\to H}\right),\\
\frac{dR}{dt} &= \sum_j Q^j(t-\tau_{Q\to R}).
\end{aligned}
$$

$$
\begin{aligned}
\frac{dS_M^i}{dt} &= -S_M\left(\frac{\beta_{MM}I_M^i+\beta_{PM}(I_P^i+H^i)+\beta_{VM}I_v^i}{N^i}+\frac{\beta_L I}{N}\right),\\
&\text{for } i\in 1,2,\dots,36,\\
\frac{dS_P^i}{dt} &= -S_P\frac{\beta_{MP}I_M^i+\beta_{PP}(I_P^i+H^i)+\beta_{VP}I_v^i}{N^i},\\
\frac{dS_V^i}{dt} &= -S_P\left(\frac{\beta_{MV}I_M^i+\beta_{PV}(I_P^i+H^i)+\beta_{VV}I_v^i}{N^i}+\frac{\beta_L I}{N}\right),\\
\frac{dE_M^i}{dt} &= S_M\left(\frac{\beta_{MM}I_M^i+\beta_{PM}(I_P^i+H^i)+\beta_{VM}I_v^i}{N^i}+\frac{\beta_L I}{N}\right)-E_M^i(t-\tau_{E\to I}),\\
\frac{dE_P^i}{dt} &= S_P\frac{\beta_{MP}I_M^i+\beta_{PP}(I_P^i+H^i)+\beta_{VP}I_v^i}{N^i}-E_P^i(t-\tau_{E\to I}),\\
\frac{dE_V^i}{dt} &= S_P\left(\frac{\beta_{MV}I_M^i+\beta_{PV}(I_P^i+H^i)+\beta_{VV}I_v^i}{N^i}+\frac{\beta_L I}{N}\right)-E_V^i(t-\tau_{E\to I}),\\
\frac{dI_M^i}{dt} &= E_M^i\left(t-\tau_{E\to I}\right)-I_M^i(t-\tau_{I\to Q}),\\
\frac{dI_P^i}{dt} &= E_P^i\left(t-\tau_{E\to I}\right)-I_P^i(t-\tau_{I\to Q}),\\
\frac{dI_V^i}{dt} &= E_V^i\left(t-\tau_{E\to I}\right)-I_V^i(t-\tau_{I\to H}),\\
\frac{dQ^i}{dt} &= I_M^i\left(t-\tau_{I\to Q}\right)+I_P^i\left(t-\tau_{I\to Q}\right)+H^i\left(t-\tau_{I\to Q}\right)-Q^i(t-\tau_{Q\to R}),\\
\frac{dH^i}{dt} &= \sum_j\left[aI_V^j\left(t-\tau_{I\to H}\right)+aI\left(t-\tau_{I\to H}\right)\right]-H^i\left(t-\tau_{H\to Q}\right).
\end{aligned}
$$

Note that the model was simulated using stochastic algorithm. The algorithm and the process of the simulation are described in the next session.

3 Stochastic model simulation

Deterministic models formulated with differential equations can be numerically solved. However, deterministic models assume the average behavior of individuals. During the early stages of an outbreak, disease propagation can be profoundly influenced by the unique characteristics of a few individuals. This can lead to occurrences of extreme variability, such as super-spreader events and cluster infections. Given this high level of uncertainty, our study primarily employs a stochastic approach. In this session, we introduce a modified Gillespie algorithm, which takes individual non-delayed and delayed events into account [7].

From the algorithm, the probability that certain Markovian process (disease transmission in this study) occurs is proportional to the value of term which represents the event, and the timing that the next event occurs (τ) is generated as $\tau \; -ln(u)/r$, where r is the sum of propensities (disease transmission terms in equations). Therefore, the next time is updated as $t + \tau$. Later, let τ^* be the most eminent timing of delayed event (non-Markovian) previously generated. The modified Gillespie algorithm compares the values of t+τ and τ^* and determines whether the next event will be nondelayed or delayed. Fig 3 provides a schematic diagram of the algorithm. In other words, if $t + \tau < \tau^*$, then the next event is Markovian, and the time is updated to $t + \tau$. If $t + \tau > \tau^*$, then the next event is non-Markovian, and the time is updated to τ^*. In each simulation, it iterates these introduced processes until every infected hosts are recovered, and we ran 10,000 times of simulations. Table 1 lists possible events during the model simulation, and Table 2 lists estimated transmission rates for the nondelayed events. Detailed description of estimating transmission rates is in the next session.

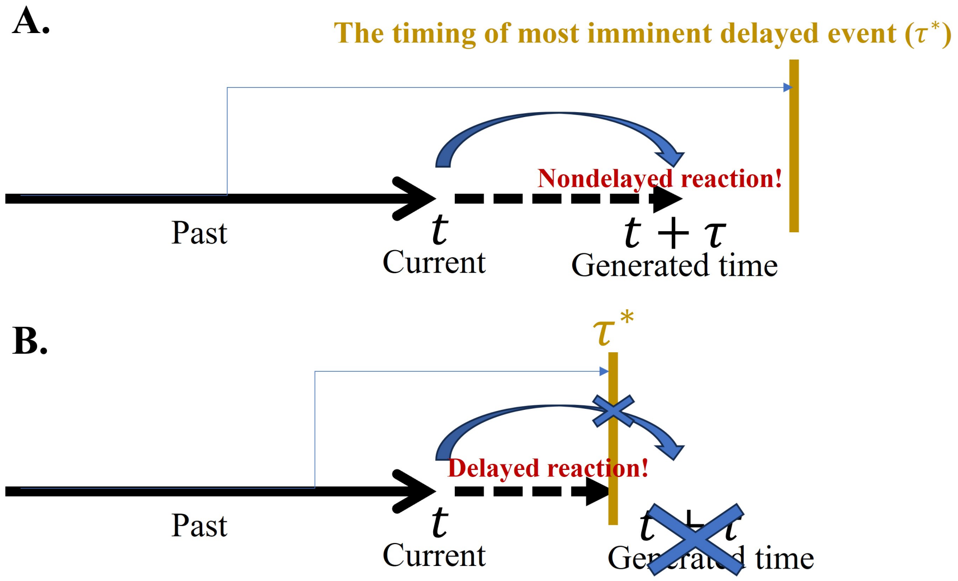

Fig 3. Two possible cases in modified Gillespie algorithm: The case when $t + \tau < \tau^*$(A), the case when $t + \tau > \tau^*$ (B). Here, t represents the current time, τ is generated time for nondelayed event, and τ^* is the most imminent delayed event.

Table 1. Characteristics of the propensity of nondelayed events and details of delayed events for stochastic simulation. These events contain estimated distributions and parameters [2, 3].

Event	Type	Description
Infection of medical staff	Nondelayed	Propensity: $S_M \left(\frac{\beta_{MM} I_M^i + \beta_{PM} (I_P^i + H^i) + \beta_{VM} I_v^i}{N^i} + \frac{\beta_L I}{N} \right)$
Infection of inpatient	Nondelayed	Propensity: $S_P \frac{\beta_{MP} I_M^i + \beta_{PP} (I_P^i + H^i) + \beta_{VP} I_v^i}{N^i}$
Infection of hospital visitor	Nondelayed	Propensity: $S_P \left(\frac{\beta_{MV} I_M^i + \beta_{PV} (I_P^i + H^i) + \beta_{VV} I_v^i}{N^i} + \frac{\beta_L I}{N} \right)$
Infection of local resident	Nondelayed	Propensity: $S \frac{\beta_L \left(I + \sum_j \left(I_M^j + I_V^j \right) \right)}{N}$
From exposure to onset	Delayed	Gamma distribution, Mean: 6.99, SD: 3.31
Symptom onset to isolation or hospitalization	Delayed	Gamma distribution, Mean: 5.53, SD: 3.70
Isolation to recovery	Delayed	Constant delay, 28 days

Table 2. Estimated transmission rates for the model simulation [3].

**Symbol**	**Description**	**Value (95% CI)**
β_L	Transmission rate in local community	0.1
β_{PM}	Transmission rate to medical staff by inpatient	0.16, [0.01, 0.47]
β_{PP}	Transmission rate among inpatients	1.40 [0.84, 2.07]
β_{PV}	Transmission rate to hospital visitor by inpatient	0.49 [0.26, 0.79]
β_{VM}	Transmission rate to medical staff by visitor	0.69 [0.03, 1.88]
β_{VP}	Transmission rate to inpatient staff by visitor	0.59 [0.02, 2.06]
β_{VV}	Transmission rate among hospital visitors	0.15 [0.00, 0.56]

For the model simulation, we focused on the Gangnam District in Seoul, Korea, known for its advanced medical infrastructure. The district comprises two tertiary referral hospitals (mean bed capacity: 1,533), two general hospitals (mean bed capacity: 225), and 32 smaller hospitals (mean bed capacity: 73) [4]. In total, there are 9,305 medical staffs and 5,222 inpatients. Based on epidemiological investigations conducted in major Gangnam District hospitals, we assumed that the number of hospital visitors equals the number of inpatients [5]. The population of the local community is 650,000 [6]. Notably, we excluded smaller clinics from our analysis. Our model simulation commenced with the introduction of a single primary case into the local community. To align with the Korean context in 2015, non-pharmaceutical interventions began six days after the admission of the index case, coinciding with outbreak recognition. These interventions restrict hospital visitors and reduce transmission rates within the hospital, resulting in a 41.02% reduction. Detailed methods for estimating transmission rates and this reduction are presented in the subsequent section.

4 Metropolis-Hastings parameter sampling

Kim's study yielded valuable insights into individual hosts, considering their host type (medical staff, patients, visitors). The analysis encompassed expected exposure (and contagious) periods, isolation times, and the number of individuals at Pyeongtaek St. Mary's Hospital (PMH) [3]. We denote the transmission rate as β_{AB}, where subscripts

A and B correspond to infector and infectee types, respectively. Subscripts indicate host categories: medical staff, patients, and hospital visitors. Subsequently, we categorized individuals as either infected or uninfected. The group of infected individuals is denoted by Λ_I, while the uninfected group is represented as Λ_S. We introduced identifiers D_i and $\tilde{D}_i$ to distinguish the host types within Λ_S and Λ_I, respectively. Additionally, let T and T^* denote the discrete time points before the outbreak recognition and after outbreak recognition (6 days after the introduction of the first infector) [3]. Given the count of infectors at a specific time k denoted as $\mathbf{I}_j(k)$, where subscript j indicates the host type, we derive the likelihood of hosts in Λ_S and Λ_I as follows:

$$\begin{aligned}
L_S &= \prod_{i \in \Lambda_S} \left\{ \prod_{k \in T} \exp\left(\sum_{j \in \{M,P,V\}} -\beta_{jD_i} \frac{\mathbf{I}_j(k)}{N} \right) \right\}, \\
L_S^* &= \prod_{i \in \Lambda_S} \left\{ \prod_{k \in T^*} \exp\left(\sum_{j \in \{M,P,V\}} -(1-q)\beta_{jD_i} \frac{\mathbf{I}_j(k)}{N} \right) \right\}, \\
L_I &= \prod_{i \in \Lambda_I} \left\{ \prod_{k \in T} \left(1 - \exp\left(\sum_{j \in \{M,P,V\}} -\beta_{j\tilde{D}_i} \frac{\mathbf{I}_j(k)}{N} \right) \right) \right\}, \\
L_I^* &= \prod_{i \in \Lambda_I} \left\{ \prod_{k \in T^*} \left(1 - \exp\left(\sum_{j \in \{M,P,V\}} -(1-q)\beta_{j\tilde{D}_i} \frac{\mathbf{I}_j(k)}{N} \right) \right) \right\}, \\
L(B) &= L_S \times L_S^* \times L_I \times L_I^*, \\
\text{where } B &= \{\beta_{MM}, \beta_{MP}, \beta_{MV}, \beta_{PM}, \beta_{PP}, \beta_{PV}, \beta_{VM}, \beta_{VP}, \beta_{VV}, q\}.
\end{aligned}$$

Note that the number of infectious individuals in certain period, $\mathbf{I}_j(k)$, was aggregated from Kim's study. Based on the epidemiological investigation, we counted the number of infectious individuals during a specific period. Our objective is to determine the optimal value of B that maximizes the likelihood function $L(B)$. Notably, we set transmission rates β_{MM}, β_{MP}, and β_{MV} to zero based on epidemiological investigations, which revealed no contagious period involving medical staff at PMH. We assumed symmetric transmission between patients and visitors, that is, $\beta_{PV} = \beta_{VP}$. Furthermore, to capture the reduction in transmission after outbreak recognition, we applied a reduction factor q to all transmission rates immediately following recognition. To sample the parameters and estimate the distributions of transmission rates, we employed the Metropolis-Hastings algorithm [8].

The Metropolis-Hastings algorithm is a Markov chain Monte Carlo method used to estimate the distribution of unknown parameters. It serves as a technique to generate random samples from a probability distribution that may be challenging to sample directly. It works by constructing a Markov chain that has the desired distribution as its stationary distribution. The algorithm commences with an initial value and iteratively generates subsequent values. This is done by proposing a candidate value from a proposal distribution and deciding to accept or reject it based on an acceptance probability. This probability depends on the ratio of the target distribution to the proposal distribution at the current and proposed values. We assumed the presence of Gaussian additive noise and a uniform prior distribution and sampled parameters in the following manner:

At iteration i with x_i,

1. Generate x^* from a proposal distribution, $x^*\ q(x_i)$, q is Gaussian
2. Accept or reject with acceptance rate $\alpha = min\big[1, \frac{f(x^*|Y)}{f(x_i|Y)}\big]$

In our study, the likelihood function is $f(x_i|Y) = \int P(Y|x,\sigma_i)P(\sigma_i)d\sigma_i \propto F(x_i)$,, where $F(x)$ is the target function to maximize, i.e., likelihood we formulated, and P

symbolizes the prior. We sampled 400,000 points and excluded 100,000 burn-in samples, which refers to the initial set of samples that we discard because they might be biased by the initial conditions, resulting in 300,000 samples considered per parameter.

Furthermore, we intentionally set the transmission rate in the local community (β_L) to 0.1, which is lower than the transmission rate within the hospital. This choice implies a basic reproduction number of approximately 0.5 in the local community. Notably, in Korea, only one infection case occurred outside the hospital among all reported cases [9].

Fig 4 visualizes the traces of parameter samples using the Metropolis-Hastings algorithm, with overlapping traces appearing more intense in color. The red curves indicate the burn-in samples, and we consider the distribution of the samples from the dark curves (histogram plots next to their traces). Panel A to F illustrates the transmission rates within the hospital. In Panel G, we present the reduction in transmission rates following outbreak recognition. Specifically, when the infector is a patient (β_{PX}), the values are estimated as mean 0.16 (95% confidence interval (CI) [0.01, 0.47]), 1.40 (95% CI [0.84, 2.07]), and 0.49 (95% CI [0.26, 0.79]) when the infectee is medical staff (β_{PM}), patient (β_{PP}), and visitor (β_{PV}), respectively. When the infector is a visitor (β_{VX}), the values are estimated as mean 0.69 (95% CI [0.03, 1.88]), 0.59 (95% CI [0.02, 2.06]), and 0.15 (95% CI [0.00, 0.56]) when the infectee is medical staff (β_{VM}), patient (β_{VP}), and visitor (β_{VV}), respectively. The reduction in the infection transmission rate after outbreak recognition (q) is an average of 41.02% (95% CI [19.88, 72.28]). In our simulations, we employed the mean values of the estimated posterior distributions for each transmission rate parameter.

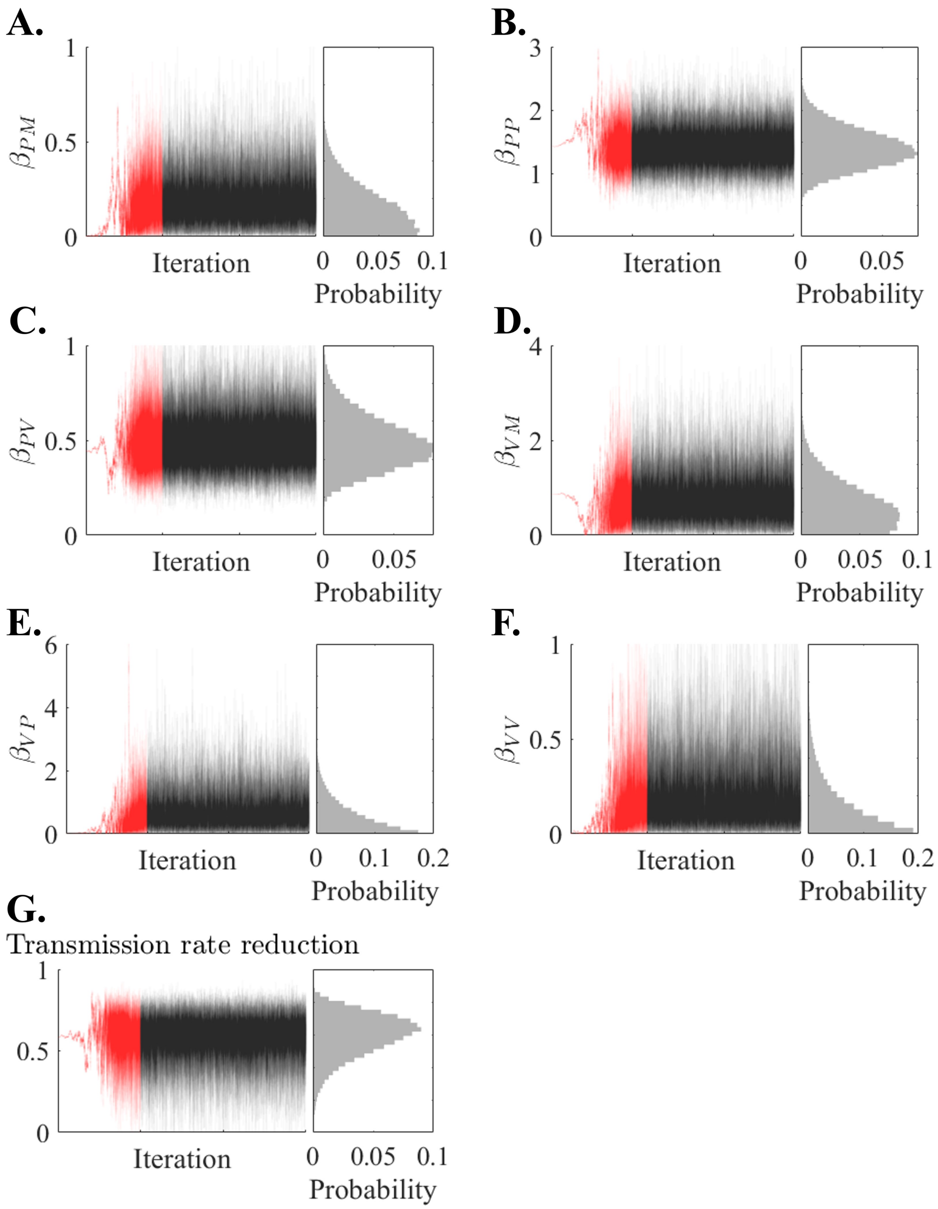

Fig 4. Traces and distributions of sampled parameters using Metropolis-Hastings algorithm. For traces, the red curves indicate the burn-in samples, while the dark curves represent the trace of samples used in this study.

References

1. Slayton RB, O'Hagan JJ, Barnes S, Rhea S, Hilscher R, Rubin M, Lofgren E, Singh B, Segre A, Paul P. Modeling infectious diseases in healthcare network (MInD-Healthcare) framework for describing and reporting multidrug-resistant organism and healthcare-associated infections agent-based modeling methods. Clinical Infectious Diseases. 2020 Nov 1;71(9):2527-32.

2. Virlogeux V, Park M, Wu JT, Cowling BJ. Association between severity of MERS-CoV infection and incubation period. Emerg Infect Dis. 2016;22(3):526.

3. Kim KM, Ki M, Cho SI, Sung M, Hong JK, Cheong HK, Kim JH, Lee SE, Lee C, Lee KJ, Park YS. Epidemiologic features of the first MERS outbreak in Korea: focus on Pyeongtaek St. Mary's Hospital. Epidemiology and health. 2015;37.

4. Nationwide status of hospitals and pharmacies [Internet], Health Insurance Review & Assessment Service. Available from: `https://opendata.hira.or.kr/op/opc/selectOpenData.do?sno=11925&publDataTpCd=&searchCnd=&searchWrd=%EC%A0%84%EA%B5%AD&pageIndex=1`

5. Cho SY, Kang JM, Ha YE, Park GE, Lee JY, Ko JH, Lee JY, Kim JM, Kang CI, Jo IJ, Ryu JG. MERS-CoV outbreak following a single patient exposure in an emergency room in South Korea: an epidemiological outbreak study. The Lancet. 2016;388(10048):994-1001.

6. Population by administrative district (city, county, district) and gender [Internet]. Ministry of the Interior and Safety. Available from: `https://kosis.kr/statHtml/statHtml.do?orgId=101&tblId=DT_1B040A3`

7. Anderson DF. A modified next reaction method for simulating chemical systems with time dependent propensities and delays. The Journal of chemical physics. 2007;127(21).

8. Chib S, Greenberg E. Understanding the metropolis-hastings algorithm. The american statistician. 1995;49(4):327-35.

9. Ki M. 2015 MERS outbreak in Korea: hospital-to-hospital transmission. Epidemiology and health. 2015;37.
