# Supplementary material for: Stochastic modeling of intra- and inter-hospital transmission in Middle East respiratory syndrome outbreak
Source: PLoS One. 2025 Sep 26;20(9):e0332116. doi: 10.1371/journal.pone.0332116 (PMC12469155; doi:10.1371/journal.pone.0332116)
Supplement: Attachment — Submitted filename: Korea_MERS_review.docx [file pone.0332116.s002.docx]

The article “Stochastic Modeling of Intra- and Inter-Hospital Transmission in Middle East Respiratory Syndrome Outbreak” models the spread of MERS within hospitals and the general population in the Gangnam District of Seoul, Korea. The authors use the model to predict outputs such as the size of the outbreak, number of exposed hospitals, and outbreak duration and assess the effect of mask mandates on the results. I thought the article was very well written, the methodology sound, and interpretation reasonable. My comments are generally minor and given below:

In the main manuscript:

- L101 and elsewhere: I think 95% prediction interval may be a more appropriate term for estimates from simulated output as opposed to credible interval

In the supplementary materials:

- L11: The infectious state is missing from the list of state variables
- L68: Please specify the gamma distribution parameters. Are they given as the shape and scale, or the shape and rate?
- L74: I recommend defining the different transmission rate parameters ($\beta_{L}, \beta_{MM}$, etc.) before their first appearance in the differential equations
- L74-75: Note that the differential equations for the infectious period are given twice ($\frac{dI_{M}^{i}}{dt}$, $\frac{dI_{P}^{i}}{dt}$, $\frac{dI_{V}^{i}}{dt}$) and that the two equations for $\frac{dI_{V}^{i}}{dt}$ differ from one another. There also seems to be a typo in the $\frac{dI_{V}^{i}}{dt}$ equation where $E_{M}^{i}$ should be $E_{V}^{i}$.
- L74-75: What does *a* represent in $\frac{dH^{i}}{dt}$?
- Table 1: Is there also a delay between hospitalization and placement in quarantine for visitors and the local population? How was this modeled?
- Table 2: What inputs were used in the model for the transmission rates? Did you use the modes from the posterior distributions?
- L169-171: It’s unclear to me how the reduction in the infection transmission rate after outbreak recognition was estimated.
