# Supplementary material for: Stochastic modeling of intra- and inter-hospital transmission in Middle East respiratory syndrome outbreak
Source: PLoS One. 2025 Sep 26;20(9):e0332116. doi: 10.1371/journal.pone.0332116 (PMC12469155; doi:10.1371/journal.pone.0332116)
Supplement: Attachment — Submitted filename: Response to Reviewers.docx [file pone.0332116.s003.docx]

**Reviewer #1**

**Comment R1-1**

Introduction:

The background related to Gangnam District in Seoul, Korea, and the two surveyed hospitals should be mentioned here (population study)

**Response R1-1**

Thank you for your suggestion. We have moved the background information on Gangnam District and the two surveyed hospitals to the Introduction section (L41–45).

###################################################################################

**Comment R1-2**

- Methods:

o Add information on the sampling methodology

o Inclusion/exclusion criteria

o List and define variables to be analyzed

o The various scenarios and their sequence should be indicated, from baseline to other models

o The sensitivity analysis to be mentioned as well

**Response R1-1**

**-Add information on the sampling methodology-**

We have added following paragraph in the manuscript considering your comment (L97-102).

“*Parameter estimation was performed using the Metropolis-Hastings algorithm. We employed stochastic simulation based on a modified Gillespie algorithm, incorporating both Markovian (non-delayed) and non-Markovian (delayed) processes. Each simulation was initiated with the introduction of a single index case into the local community. For each scenario, we performed 10,000 independent simulation runs to capture the stochastic variability in transmission dynamics. Detailed description is in S1 Text.”*

**-Inclusion/exclusion criteria-**

We have added following paragraph in the manuscript considering your comment (L54-62).

*“Our model includes individuals who are either hospitalized or directly interacting with hospital settings in the Gangnam District, Seoul. Specifically, the included populations are inpatients, hospital visitors, and medical staff across 36 hospitals (two tertiary, two general, and 32 smaller hospitals). The simulation is initiated with the importation of a primary case into the local community (population: 650,000), and transmission dynamics are limited to hospital-related settings. We excluded small clinics that do not accommodate inpatients, due to lack of transmission data and limited epidemiological relevance. Additionally, we did not include individuals from the general community who had no hospital exposure during the outbreak.”*

**-List and define variables to be analyzed-**

We have added following paragraph in the manuscript considering your comment (L63-75).

*“We analyzed the following key outcome variables and the role of uncertainty-from the stochastic simulations:*

- *Total number of individuals who became infected during the simulated outbreak.*
- *Number of hospitals with at least one infected individual (i.e., where any exposed, infectious, hospitalized, or isolated individual is present).*
- *Time elapsed from symptom onset of the index case to the isolation of the last infected individual.*
- *Maximum number of individuals in the isolated compartment at any single point in time.*
- *Daily number of individuals in exposed, infectious, and isolated states throughout the simulation.*
- *Sensitivity analysis metric to quantify the influence of input parameters on the total number of infections.”*

**-The various scenarios and their sequence should be indicated, from baseline to other models-**

We have added following paragraph in the manuscript considering your comment (L104-116).

*“We designed a series of simulation scenarios to assess outbreak dynamics under varying conditions. The scenarios are described below in sequential order:*

1. *Baseline scenario: The index case is introduced into the community, and the outbreak is recognized 5 days after hospital admission. Hospital visits are prohibited at recognition, and intra-hospital transmission is reduced by 59%. Upon symptom onset, a host is randomly admitted to a hospital.*
2. *Outbreak detection delay: We varied the detection delay from 1 to 28 days to evaluate the risk of late outbreak recognition.*
3. *Mask intervention: We simulated the impact of intra-hospital mask mandates, assuming reduction effects in transmission ranging from 0% (baseline) to 80%.”*

**-The sensitivity analysis to be mentioned as well-**

We have added following paragraph in the manuscript considering your comment (L133-146).

*“Lastly, we performed a sensitivity analysis to identify the key factors influencing the outbreak. Using Latin Hypercube Sampling, we calculated the Partial Rank Correlation Coefficients (PRCC) for each input parameter [28]. The model incorporated several input variables: the timing of outbreak detection (τ_rec), intra-hospital transmission rates (β_H), the infectious period within hospitals for the primary case (τ̃_I→Q), the infectious period within hospitals for non-primary cases (τ_I→Q), the transmission rate in the local community (β_L), the infectious period in the community for the primary case (τ̃_I→H), the infectious period for other local cases (τ_I→H), the incubation period (τ_E→I), and the number of hospital visitors (S^i_V)}. A total of 200,000 simulation runs were conducted, with each parameter varied uniformly by ±30% from its baseline value. The intra-hospital transmission rate parameter (β_H) collectively represented all transmission rate parameters within the hospital. Delay-related parameters were adjusted based on distributions generated during the model simulation. The primary output of interest was the cumulative number of infections, measured as the total number of individuals transitioning from the susceptible to exposed state.”*

**Comment R1-3**

- Results:

The results for each model should be described more systematically, and summarized. What has changed between the baseline model and the other model?

**Response R1-3**

We have restructured the *Results* section by adding a brief overview and summary to clearly compare the baseline scenario with other scenarios.

**(L148-154)**

*“We examined multiple scenarios to evaluate the impact of outbreak detection timing and hospital-based preventive interventions on nosocomial transmission dynamics. Specifically, we compared a baseline scenario—representing moderate early detection and no mask mandates—with alternative scenarios involving delayed detection (ranging from 1 to 28 days), mask mandates with varying effectiveness (0% to 80%), and their combined effects. Each scenario was simulated using a stochastic framework to assess variability in outbreak size, duration, and peak burden.”*

**(L215-221)**

***“Summary of scenario comparisons.*** *Compared to the baseline scenario, delayed outbreak detection led to substantial increases in outbreak size and isolation burden. However, when moderate to high mask effectiveness (≥60%) was assumed, these effects were largely mitigated. The combined implementation of early detection and mask mandates was the most effective in reducing peak isolation burden. Overall, these results emphasize the critical role of timely outbreak recognition and intra-hospital interventions in minimizing outbreak impact.”*

**Comment R1-4**

- Discussion:

Each result should be discussed more systematically and following the order of appearance in the results section

- Strengths and Limitations of this study should be indicated separately

- A section on Conclusion is to be added separately

- A separate section on Recommendations to be added as well

**Response R1-4**

**- Strengths and Limitations of this study should be indicated separately**

We have revised the manuscript as follows (L269-272):

*“One strength of this study is its flexible framework for evaluating multiple policy scenarios using stochastic modeling that reflects real-world delays and heterogeneity. Our use of epidemiologically-derived parameters and detailed simulation design enables realistic assessment of hospital-level transmission dynamics and control strategies.”*

- A section on Conclusion is to be added separately

We have added conclusion section as follows (L286-293):

*“Our findings highlight the critical role of early outbreak recognition and targeted hospital-level interventions in controlling nosocomial MERS transmission. Even under delayed detection, effective mask mandates can significantly reduce peak burden, underscoring their importance as a first-line defense in healthcare settings.”*

- A separate section on Recommendations to be added as well

We have added recommendation part (L296-305):

*“Based on our findings, we recommend prioritizing early outbreak recognition through streamlined diagnostic workflows in hospitals. Our simulation results indicate that even modest delays in detection can substantially increase outbreak size and isolation burden. In particular, detection delays beyond two weeks may exceed local isolation capacity unless mitigated by additional interventions.*

*We also recommend maintaining mask mandates within hospital settings during periods of emerging respiratory threats. Our results show that mask effectiveness of 60\% or higher can partially compensate for delayed detection and significantly reduce peak isolation burden. Finally, maintaining a baseline level of surge capacity for isolation beds in densely populated urban areas may help buffer against sudden outbreak escalation.”*

**Reviewer #2**

**Comment R2-1**
The materials and methods are too shallow and hard to comprehend. Introduce key steps in this section and describe them concisely. For example, the statistical methods used in assessing correlations between correlation exists between confirmed cases and exposed hospitals need to be described among others.

**Response R2-1**

We have revised the Methods section to describe our workflow step by step (L54-75, L97-102). As the correlation between confirmed cases and exposed hospitals was assessed using Pearson’s correlation coefficient—a widely used and straightforward statistic—we opted to introduce it succinctly in the Results rather than in extensive detail. Accordingly, we added the following sentence (L167-169):

*“To quantify the relationship between the total number of confirmed cases and the number of exposed hospitals across simulation runs, we calculated Pearson’s correlation coefficient.”*

###################################################################################

**Comment R2-2**

In materials and methods, you mention that your analysis ignored smaller clinics. Much as this could be a simplifying assumption, it may impact the study outcomes. Investigate (as a sensitivity analysis) how their inclusion would affect the study outcomes.

**Response R2-2**

In the revised manuscript, we have clarified in our Limitations section that smaller clinics were excluded because they overwhelmingly lack inpatient services and reliable transmission data, and because incorporating thousands of such facilities would exceed the capabilities of our current stochastic framework. We note that their inclusion would be best handled via an agent-based modeling approach, which we intend to pursue in future research (L281-284).

*“We excluded small clinics because they lack inpatient data and modeling thousands of tiny facilities exceeds our stochastic framework; we will address this in future agent‐based work.”*

**Comment R2-3**

For improved content flow, some key informative details on model building and parameterization need to be (concisely) introduced in the main text instead of supplementary material. E.g., in line 83, on how was the value of 59% arrived? This and other similar mentions need description instantly. You can keep the detailed descriptions about the same parameters in the supplementary file.

**Response R2-3**

Thank you for catching this error. We discovered a typo—the intra-hospital transmission reduction should be 41.02%, not 59%—and have corrected it in the main text. We have also added a brief statement in the Methods to explain that this value was estimated using Metropolis-Hastings parameter sampling (see Supplementary Text S1 for full details).

###################################################################################

**Comment R2-4**

How realistic is the assumption of 0% mask use impact in the baseline scenario? It is a known fact that irrespective of the prevailing circumstances, some people would be using masks anyway. This need to be revised.

**Response R2-4**

Thank you for this point. In 2015 Korea, awareness and use of masks in hospitals were minimal, and in fact hospital visitation was very common. Moreover, our baseline scenario already incorporates a reduction in transmission rates within hospitals following outbreak recognition, independent of mask use. To make this explicit, we have now added a clear statement in the main text that the baseline assumes 0% mask effectiveness (L104-108).

*“In the baseline scenario we assume 0% mask effectiveness—reflecting the low awareness and minimal mask use in Korean hospitals during the 2015 outbreak—while the observed reduction in intra-hospital transmission is modeled solely through post-recognition interventions.”*

###################################################################################

**Comment R2-5**

What informed your choices for the ranges explored in the alternative scenarios explored? Justification should be added to the main text and practicality and feasibility should be key. You cite [28] on 80% the second scenario, (concisely and briefly) describe how they came up with that number in the main text.

**Response R2-5**

Thank you for this comment. We have now added a concise justification in the main text, noting that our 80% upper bound for mask effectiveness is informed by the pooled estimates in Offeddu et al.’s systematic review (risk ratios 0.13–0.59, corresponding to 41–87% reduction), which supports a maximal protective effect of approximately 80%. We added description into the manuscript as follows (L131-132):

*“Note that based on Offeddu et al.’s meta-analysis (risk ratios 0.13–0.59, corresponding to 41–87% reduction in infection), we set the maximum mask effectiveness at 80%”*

**Comment R2-6**

The code used for the sensitivity analysis should be provided as part of the supplementary information files.

**Response R2-6**

Thank you for the suggestion. We will make all code used for the sensitivity analysis publicly available on Figshare and provide the corresponding DOI.

###################################################################################

**Comment R2-7**

Rearrange your contact to ensure that content appears where it is most suited. Case in point is the description on sensitivity analysis (in lines 121-132) that you introduce in the results section yet it is methods.

**Response R2-7**

Thank you for the feedback. We have relocated the detailed description of the sensitivity analysis from the Results section to the Materials and Methods section (L133-146) for greater clarity.

###################################################################################

**Comment R2-8**

Tense and grammar edits are needed e.g. in abstract where you write “Our findings emphasized…” shouldn’t it be “emphasize”?

**Response R2-8**

Corrected.

###################################################################################

**Comment R2-9**

Line 14: replace “singular” with “single”

**Response R2-8**

Corrected.

###################################################################################

**Comment R2-10**

Line 47: delete “of”

**Response R2-8**

Corrected.

**Comment R2-11**

Under materials and methods, separate data description and model description in the first subheading.

**Response R2-11**

Thank you for this suggestion. As most data details are covered in the Supplementary Text, we have kept the main Materials and Methods concise. Instead, we have added an overarching overview of our methodological approach and a clear list of the key outcome variables (L54-75) to improve the organization without duplicating supplementary content.

*“Our model includes individuals who are either hospitalized or directly interacting with hospital settings in the Gangnam District, Seoul. Specifically, the included populations are inpatients, hospital visitors, and medical staff across 36 hospitals (two tertiary, two general, and 32 smaller hospitals). The simulation is initiated with the importation of a primary case into the local community (population: 650,000), and transmission dynamics are limited to hospital-related settings. We excluded small clinics that do not accommodate inpatients, due to lack of transmission data and limited epidemiological relevance. Additionally, we did not include individuals from the general community who had no hospital exposure during the outbreak.* *We analyzed the following key outcome variables and the role of uncertainty-from the stochastic simulations:*

- *Total number of individuals who became infected during the simulated outbreak.*
- *Number of hospitals with at least one infected individual (i.e., where any exposed, infectious, hospitalized, or isolated individual is present).*
- *Time elapsed from symptom onset of the index case to the isolation of the last infected individual.*
- *Maximum number of individuals in the isolated compartment at any single point in time.*
- *Daily number of individuals in exposed, infectious, and isolated states throughout the simulation.*
- *Sensitivity analysis metric to quantify the influence of input parameters on the total number of infections.”*

###################################################################################

**Comment R2-12**

Line 68: wrong phrasing in “To simulate our model…”. It is not the model that you are simulating rather it is the disease spread that you are simulating using the model

**Response R2-12**

We correct the phrase as

“*To simulate disease spread, we aggregate recorded time delays and incorporate them into the model.”*

###################################################################################

**Comment R2-13**

Line 97: use “distribution” instead of “distributions”?

**Response R2-13**

Thank you for the comment. Because we derive and sample from multiple distributions of simulation outcomes, we retain the plural form “distributions” to accurately reflect that more than one distribution is visualized.

**Comment R2-14**

Check and revise the flow of the sentence in line 98.

**Response R2-14**

We have revised the paragraph as follows:

*“We define an exposed hospital as one that has at least one infected host (E, I, or H). Outbreak duration is defined as the time from symptom onset of the index case to the isolation of the last infected host. “*

###################################################################################

**Comment R2-15**

Line 146: what are those two factors? Mention them explicitly or improve sentence flow.

**Response R2-15**

We have changed the word to ‘the outbreak detection delay and the effectiveness of mask mandates within hospitals’ for better clarity.

**Reviewer #3**

**Comment R3-1**
The authors must provide the complete code to reproduce all model fitting procedures and simulation output presented in the manuscript.

**Response R3-1**
As requested, we have uploaded the complete code for the Metropolis–Hastings parameter fitting, the stochastic simulation procedures, and sensitivity analysis to Figshare (DOI: https://doi.org/10.6084/m9.figshare.26317555.v3).

###################################################################################

**Comment R3-2**
There is already a substantial modeling study of the MERS. What is the unique contribution of this work, and how does the model offer meaningful insights or practical relevance in the current context?

**Response R3-2**
In this study, we specifically model transmission within healthcare facilities—a distinctive cluster type compared to community or small‐cluster settings. While mask use in hospitals is now common post–COVID-19, during the 2015 MERS outbreak in Korea, hospital visits and lack of mask wearing were prevalent. Our work demonstrates how hospital‐level mask mandates can meaningfully reduce nosocomial spread and prevent subsequent community transmission. We added following paragraph into the manuscript (L286-289):

*“Our model focuses on both of inter- and intra‐hospital transmission dynamics. Reflecting the minimal mask use and active visitation culture during the 2015 Korean MERS outbreak, we quantify how hospital‐based mask mandates can both curb nosocomial spreads and avert broader community outbreaks.”*

**Comment R3-3**
The study assumed that the number of hospital visitors equals to the number of inpatients. Is there a reference to support this assumption? In addition, a sensitivity analysis exploring how variations in the ratio of hospital visitors to inpatients affect the model outcomes should be conducted and included.

**Response R3-3**
We based our assumption that the number of inpatients and visitors are similar on data from Samsung Medical Center (patient-to-visitor ratio = 675:683). The corresponding section in the Appendix has been updated as follows (L111-114).

*“Based on epidemiological investigations conducted in major Gangnam District hospitals, we assumed that the number of hospital visitors equals the number of inpatients.”*

We also included the visitor-to-inpatient ratio in the sensitivity analysis; its PRCC ranged from –0.01 to 0.02, indicating a minimal impact on outbreak outcomes (L190-192).

*“Additionally,* $\tau_{I\to H}$ *and*$S_{V}^{i}$ *exhibited PRCC of 0.01 to 0.02 and –0.01 to 0.02, respectively, indicating a negligible effect on outbreak outcomes.”*

**Comment R3-4**
In Figure 1, which presents a diagram of the model, the solid arrows represent the pathways of the infection transmission. However, it is unclear what the dotted arrows and red dotted arrows indicate. Additionally, both I_V^i and I flow into “red (2)”, but it is unclear what this “red (2)” signifies. Is it not a specific compartment? Why was it depicted in red?

**Response R3-4**
Thank you for pointing this out. The red arrows were originally used to distinguish transfers of infected individuals to other hospitals. To avoid confusion, we have updated the figure and changed these red arrows to black.

###################################################################################

**Comment R3-5**
The equations in the Supporting Information (S1 text) need to be organized, as some equations are repeated. Furthermore, based on the diagram in Figure 1, I_M^i, I_P^i, and H^i flow into Q^i. However, the equations state (dQ^i)/dt=I_M^i (t-τ_(I→Q) )+I_P^i (t-τ_(I→Q) )+I_V^i (t-τ_(I→Q) )-Q^i (t-τ_(Q→R)). The author could provide a more detailed explanation or clarification.

**Response R3-5**
Thank you for catching this typo. We have corrected the equation as follows:

which now fully aligns with the flow diagram.

**Reviewer #4**

**Comment R4-1**

L101 and elsewhere: I think 95% prediction interval may be a more appropriate term for estimates from simulated output as opposed to credible interval

**Response R4-1**
We corrected Credible interval (CrI) into Prediction interval (PI).

###################################################################################

**Comment R4-2**

SL11: The infectious state is missing from the list of state variables

**Response R4-2**
Corrected.

###################################################################################

**Comment R4-3**

L68: Please specify the gamma distribution parameters. Are they given as the shape and scale, or the shape and rate?

**Response R4-3**
The parameters are shape and scale parameter. We clarify it as follows (L68-69):

“Note that estimated parameters for the distributions are scale and shape parameters.”

###################################################################################

**Comment R4-4**

L74: I recommend defining the different transmission rate parameters ($\beta_{L}, \beta_{MM}$, etc.) before their first appearance in the differential equations

**Response R4-4**
Thank you for your suggestions. We added the following paragraphs before the equations (L79-81).

*“The transmission rate in the community is denoted by β_L, and within hospitals the subscripts indicate the infector and infectee, respectively. For example, β_{PV} represents the transmission rate from inpatients to visitors.”*

**Comment R4-5**

L74-75: Note that the differential equations for the infectious period are given twice ($\frac{dI_{M}^{i}}{dt}$, $\frac{dI_{P}^{i}}{dt}$, $\frac{dI_{V}^{i}}{dt}$) and that the two equations for $\frac{dI_{V}^{i}}{dt}$ differ from one another. There also seems to be a typo in the $\frac{dI_{V}^{i}}{dt}$ equation where $E_{M}^{i}$ should be $E_{V}^{i}$.

**Response R4-5**

Thank you for catching this typo. We have corrected the equation as follows:

###################################################################################

**Comment R4-6**

L74-75: What does *a* represent in $\frac{dH^{i}}{dt}$?

**Response R4-6**

We have clarified in the text that the parameter ‘a’ represents the uniform probability that an infected visitor is admitted to any one of the 36 hospitals. In other words, a=1/36. We revised the supplementary appendix as follows (L74-77):

*“If visitors become infected, they are randomly assigned to one of the hospitals—potentially different from their original point of contact. To model this, we include the parameter a, defined as the inverse of the number of hospitals in our study (a=1/36), which uniformly distributes incoming infected visitors across all facilities.”*

**Comment R4-7**

Table 1: Is there also a delay between hospitalization and placement in quarantine for visitors and the local population? How was this modeled?

**Response R4-7**

Both visitors and individuals in the local community experience two sequential delays: (1) from infection onset to hospital admission, and (2) from hospital admission to quarantine. In our model, these delays are drawn from the same fitted distribution for the time from infectious onset to isolation. We have added the following clarification to the main text (L63-65):

*“Both visitors and local residents undergo two delays: infection‐to‐hospital admission and admission‐to‐quarantine. Each delay is sampled from the same gamma distribution fitted to the observed time from symptom onset to isolation.”*

###################################################################################

**Comment R4-8**

Table 2: What inputs were used in the model for the transmission rates? Did you use the modes from the posterior distributions?

**Response R4-8**

We used the posterior means for all transmission rate parameters. To clarify this in the manuscript, we have added the following sentence (L183-184):

*“In our simulations, we employed the mean values of the estimated posterior distributions for each transmission rate parameter.”*

**Comment R4-9**

L169-171: It’s unclear to me how the reduction in the infection transmission rate after outbreak recognition was estimated.

**Response R4-9**

In our likelihood framework, we accounted for the six-day delay to outbreak recognition observed in the 2015 hospital cluster by multiplying all transmission rates by a reduction factor beginning six days after admission. To clarify, we added the following sentence (L131-133 and L143-144):

*“let T and T* denote the discrete time points before the outbreak recognition and after outbreak recognition (six days after the introduction of the first infector) [3]”*

*“Furthermore, to capture the reduction in transmission after outbreak recognition, we applied a reduction factor q to all transmission rates immediately following recognition.”*

We also modified the formulation of the likelihood function to distinguish the recognition timing as follows (L135):
